# Bio-Inspired Energy-Efficient Cluster-Based Routing Protocol for the IoT in Disaster Scenarios

**DOI:** 10.3390/s24165353

**Published:** 2024-08-19

**Authors:** Shakil Ahmed, Md Akbar Hossain, Peter Han Joo Chong, Sayan Kumar Ray

**Affiliations:** 1 Department of Mechanical and Electrical Engineering, Massey University, Palmerston North 4442, New Zealand; shakil.ahmed@manukau.ac.nz; 2 School of Computing, Eastern Institute of Technology, Napier 4112, New Zealand; 3 Department of Engineering, Computers, and Mathematical Sciences, Auckland University of Technology, Auckland 1010, New Zealand; peter.chong@aut.ac.nz; 4 School of Computer Science, Taylor’s University, Subang Jaya 47500, Malaysia; sayan.ray@taylors.edu.my

**Keywords:** IoT, WSN, clustering protocol, routing protocol, energy efficiency

## Abstract

The Internet of Things (IoT) is a promising technology for sensing and monitoring the environment to reduce disaster impact. Energy is one of the major concerns for IoT devices, as sensors used in IoT devices are battery-operated. Thus, it is important to reduce energy consumption, especially during data transmission in disaster-prone situations. Clustering-based communication helps reduce a node’s energy decay during data transmission and enhances network lifetime. Many hybrid combination algorithms have been proposed for clustering and routing protocols to improve network lifetime in disaster scenarios. However, the performance of these protocols varies widely based on the underlying network configuration and the optimisation parameters considered. In this research, we used the clustering parameters most relevant to disaster scenarios, such as the node’s residual energy, distance to sink, and network coverage. We then proposed the bio-inspired hybrid BOA-PSO algorithm, where the Butterfly Optimisation Algorithm (BOA) is used for clustering and Particle Swarm Optimisation (PSO) is used for the routing protocol. The performance of the proposed algorithm was compared with that of various benchmark protocols: LEACH, DEEC, PSO, PSO-GA, and PSO-HAS. Residual energy, network throughput, and network lifetime were considered performance metrics. The simulation results demonstrate that the proposed algorithm effectively conserves residual energy, achieving more than a 17% improvement for short-range scenarios and a 10% improvement for long-range scenarios. In terms of throughput, the proposed method delivers a 60% performance enhancement compared to LEACH, a 53% enhancement compared to DEEC, and a 37% enhancement compared to PSO. Additionally, the proposed method results in a 60% reduction in packet drops compared to LEACH and DEEC, and a 30% reduction compared to PSO. It increases network lifetime by 10–20% compared to the benchmark algorithms.

## 1. Introduction

**Figure 1:**
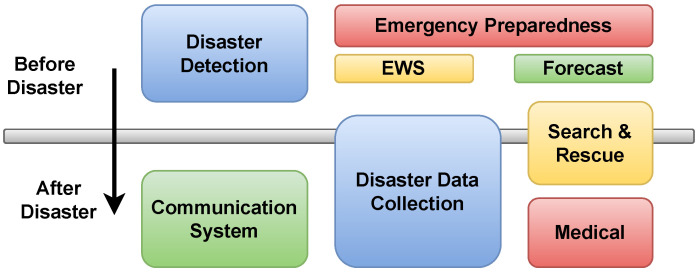
Different types of disaster applications for pre- and post-disaster scenarios.

The number of natural disasters has escalated due to global warming, environmental pollution, urbanisation, etc. According to statistics, an average of 60,000 people die from disasters each year  [1]. Faster detection of disasters can save many lives. To achieve this, it is necessary to monitor environmental parameters (temperature, wind pressure, gas or chemical compositions, etc.) by implementing a wide range of sensors. The Internet of Things (IoT) represents an emerging paradigm that provides scalable solutions for a wide range of disaster-related problems [2]. The objects (e.g., mobile phones, radio-frequency identification (RFID) tags, sensors, etc.) in IoT networks can communicate with each other to perform complex tasks by collecting and processing data without human intervention [3]. The wireless sensor network (WSN), which acts as a virtual layer of the IoT network, covers a range of IoT applications for disaster management, including pre-disaster environment monitoring [4,5], disaster preparedness [6], Early Warning Systems (EWSs) [7], and post-disaster response [8]. Figure 1 shows the WSN applications for disaster management.

In WSN-based IoT applications, sensor nodes collect data from the operating environment and send these data to a sink or base station [9], where the data are then further processed to evaluate the situation. However, there are limitations to using WSN-based IoT networks in disaster situations: limited battery life, power outages, limited memory, etc. [10]. The fundamental paradigm of these networks—such as sensing the environment, data collection, and transmission—minimises the energy level of a node, which may result in the breakdown of the communication link between the sensing nodes and the sink, disrupting the whole process. Moreover, during a disaster, replacing sensors or batteries may not be possible. As a result, the entire network may collapse, causing delays in the response system. Hence, many researchers are working towards energy-efficient communication to increase the longevity of these networks.

Cluster-based communication addresses the power-conserving issue by providing energy-efficient communication [11]. Clustering algorithms create a group of sensor nodes and decide which cluster head (CH) to associate with among various choices. In every cluster, there is a CH, and the CH is responsible for collecting data inside the cluster from nodes known as cluster members. The CH also directly manages communication with other CHs, an external gateway, or a sink. Transferring data to the base station (BS) or another CH requires high energy. Therefore, it is important to select or design an energy-efficient routing protocol to reduce the energy consumption of the CH nodes. Finding an optimal node as a CH is time-consuming and computationally expensive. Although it is an NP-hard problem, existing research shows that evolutionary algorithms can increase network efficiency [12,13,14,15,16,17]. The authors of [18] proposed an energy-efficient clustering method using a Genetic Algorithm to optimise the search for cluster heads. It considers the residual energy, the number of CHs in the network, the distance between member nodes and the CH, the distance between the CH and the sink, and the energy required to transmit data from a cluster to a sink. A similar approach was reported in [19], using a similar parameter for the fitness function, except for the weight assignment for the CH’s relative position within the cluster. In addition to the residual energy, the energy required to communicate with other nodes in the cluster and the sink was considered in [20]. However, the fitness function used for cluster formation in the above-mentioned research cannot directly apply to cluster formation in disaster scenarios. For instance, finding a cluster formation solution that supports higher scalability in a disaster scenario is necessary to re-establish communication in an affected area. Therefore, the fitness function proposed in this work considers network coverage to achieve higher scalability and other energy-related parameters.

An energy-efficient routing protocol identifies the best route to transfer data between the BS and the CH, which reduces energy consumption and enhances the lifetime of the network [21]. Several energy-efficient routing protocols have been studied to dynamically select the CH and jointly optimise the energy efficiency. However, these protocols cannot be directly applied to disaster scenarios due to sudden peak network traffic, power outages, and sensitivity to delays. Thus, this paper provides an evolutionary clustering and routing method capable of managing the energy consumption of nodes while considering the characteristics of a disaster area. Moreover, most existing works try to minimise energy consumption to increase network lifetime, whereas we consider energy efficiency to be the effective use of power for transmissions.

This paper aims to prolong network lifetime by minimising the node’s energy consumption during communication between the node and the sink. The Butterfly Optimisation Algorithm (BOA) with a modified fitness function is used for cluster formation due to its high stability and lower computational complexity. The objective of the modified fitness function is to prolong network lifetime and extend connectivity in a disaster scenario. This helps increase network lifetime. This paper proposes a bio-inspired energy-efficient clustering and routing (BICR) algorithm to enhance network lifetime in disaster scenarios. The network’s lifetime is further increased by optimising the routing path generation using Particle Swarm Optimisation (PSO). The PSO algorithm is dynamic, prompts convergence, provides the highest throughput, and reduces energy consumption compared to other heuristic and mathematical approaches. Therefore, a hybrid BOA-PSO method should increase energy efficiency more than using these techniques separately.

The main contributions of this paper are summarised as follows:

1. Proposal of an improved BOA algorithm for optimal clustering by utilising search parameters critical to a disaster scenario, including residual energy, distance to the neighbours, distance to the BS, and network coverage.
2. Proposal of a shortest path multi-hop routing protocol using the PSO algorithm. Here, PSO is optimised using residual energy, the distance between the source and the destination, and the number of relay nodes involved in the path.
3. Improvements to the state-of-the-art method to enhance average energy consumption, network coverage, and network lifetime by dynamically adjusting the sink position in different locations.

The rest of this paper is organised as follows. Section 2 highlights previous studies related to this research. Section 3 explains the network model considered in this research, while Section 4 describes the proposed CH formation with a fitness function tailored to disaster scenarios. Section 5 presents the proposed routing protocol for relaying emergency messages. The system simulation modelling and results are presented in Section 7. Finally, Section 8 concludes this paper. Table 1 lists the abbreviations used in this study.

## 2. Literature Review

It is important to note that numerous researchers have made significant contributions to the field of WSNs by proposing algorithms that enhance performance in terms of energy consumption, network coverage, and network availability. As energy is a critical factor in measuring WSN performance, extensive research has been conducted on clustering and routing algorithms to improve energy efficiency. Swarm-based optimisation methods are widely studied in the literature as one of the search strategies to find the global minimum. Finding an IoT node from a group of competitive nodes and identifying the best routing path from the source to the destination can be described as a search problem, where the objective is to minimise energy consumption and maximise network lifetime. Many researchers have focused on swarm-based methods to solve global optimisation problems, using single metaheuristic algorithms or hybrid models. This section delves into some notable previous research works, providing a comprehensive overview of existing knowledge. A plethora of optimisation algorithms have been developed that are fundamentally inspired by nature, such as the intelligent behaviours of birds, bees, fireflies, pigeons, etc. Some of the existing nature-inspired optimisation algorithms include Ant Colony Optimisation (ACO) [22], the Firefly Optimisation Algorithm (FOA) [23], Artificial Bee Colony (ABC) [24], Roach Infestation Optimisation (RIO) [25], the Cuckoo Search Algorithm (CSA) [26], Honey Bee Mating Optimisation (HBMO) [27], Particle Swarm Optimisation (PSO) [28], Pigeon-Inspired Optimisation (PIO) [29], the Bat Algorithm (BA) [30], the Lion Pride Optimiser (LPO) [31], the Butterfly Optimisation Algorithm (BOA) [32], the Lion Optimisation Algorithm (LOA) [33], and many more. ACO and PSO are extensively used for clustering and routing in WSNs.

The authors of [12,13] proposed ACO-based clustering and routing algorithms for IoT networks. The author of [12] proposed a routing algorithm using ACO to obtain the best routing benefits by categorising the IoT environment based on network types and applying the most suitable ACO to each network. Multiple network types in an IoT environment result in overlapping areas between completely different networks. However, the objective of the aforementioned algorithm is to find the optimal path between different networks, which does not consider the node’s energy level. This is critical, as it may create a network partition problem due to overuse of the path. Moreover, the performance of the algorithm significantly suffers in areas where different networks intersect each other and create an overlapping region.

To optimise routing for overlapping areas, the authors of [13] proposed a multi-agent-based ACO algorithm to avoid overlapping intersections. Four agents are considered: global, local, dual-function, and monitoring agents. The local agent is responsible for generating the routing for each IoT network. The global agent then combines this information with data from the monitoring agent to handle different networks in the IoT system. The role of the dual-function agent is to produce optimised routing for overlapping areas. However, it is still not suitable for disaster scenarios, as the fitness function of the ACO algorithm does not consider the network’s longevity.

In [19], the authors proposed a method for choosing an optimal cluster head from a group of nodes using the BOA. The fitness function of the optimiser is based on the residual energy of the nodes, the distance between nodes, the distance to the sink, the node’s degree of connectivity with others, and location. Upon selection of the CH, the route between the CH and the sink is optimised utilising ACO. The optimal path is selected based on distance, residual energy, and node degree. ACO struggles with scalability, especially in the context of large, intricate problems. As the size of the problem increases, the computational cost and the necessary iterations can grow significantly.

Multi-hop communication between the sink and the CH was considered in [34] to increase network coverage. By integrating ABC with the Firefly Algorithm, an energy-efficient cluster-based routing protocol was proposed. A self-organising map neural network is used at the beginning of cluster formation. Afterwards, the Firefly Algorithm-based optimiser adjusts the cluster size dynamically, considering the distance between the CH and the sink and the CH’s energy level. An optimised ACO algorithm is used to route the data to the base station, which can improve energy utilisation in inter-cluster communication.

The authors of [14] introduced a combined ACO- and PSO-based clustering method to optimise energy dissipation and data transmission rates. They created a fitness function based on residual energy and inter-cluster distance for data aggregation. In cluster head (CH) formation, nodes with higher residual energy are more likely to become CHs. Once a CH is selected, it uses PSO to find the best routing path (i.e., the shortest path) to the sink node. However, this method leads to congestion on the optimal path, as it only considers the Euclidean distance when building the fitness function. Moreover, PSO is also used for cluster formation. Gao et al. [35] proposed a PSO-based clustering and routing algorithm to optimise energy consumption in WSNs. The key parameters used in the PSO-based clustering algorithm are residual energy density, distance from the base station, and intra-cluster distance from the cluster head and other nodes, all aimed at extending the network’s lifetime.

The remaining energy of a node is an important parameter in forming a cluster; however, the distance to the sink and node connectivity with the neighbouring nodes can improve the performance of cluster-based communication [28]. Considering these parameters in the fitness function and using an efficient encoding scheme, the authors of [28] proposed a Harmony Search Algorithm (HSA) for clustering and routing in WSNs. It performs better than PSO- and LEACH-based routing protocols due to its mapping and effective fitness function. However, fault tolerance and delays are not considered when transmitting the information to the BS, which are critical in disaster scenarios.

An integrated algorithm using both Particle Swarm Optimisation (PSO) and the Harmony Search Algorithm (HSA) was proposed in [29]. The algorithm aims to achieve global search with a rapid convergence rate by harnessing the high computational capabilities of the HSA and dynamic movements between regions to find an optimal solution with PSO. The results indicated improved residual energy and throughput using the standalone PSO algorithm and HSA. However, no performance evaluation was conducted for network lifetime, and delays were incurred in communication. Morteza et al. [36] proposed a novel fitness function using energy efficiency, cluster closeness, and an improved PSO-HSA. The approach involves two phases: (1) an improved hybrid PSO-HSA for selecting cluster heads (CHs), optimising for energy efficiency, network closeness, and coverage using an adaptive weighted sum method, and (2) a PSO-based routing protocol with modified tree encoding to reduce invalid paths by selecting relay nodes. A new packet format includes a “fire flag” to indicate non-uniform events in disaster scenarios.

A Genetic Algorithm (GA)-based clustering and routing algorithm was presented in [30] to improve energy efficiency in WSNs. It showed sufficient improvements in energy consumption and network lifetime by using residual energy, distance, and routing phase in the fitness function. However, the network in a disaster scenario is dynamic, with rapid topology changes due to nodes joining and leaving the networks. The protocol is unable to manage such scenarios effectively. The dynamic nature of the network was considered in another GA-based algorithm [37], and an energy-efficient dynamic routing adjustment was proposed using a virtual grid concept. The method requires fewer iterations to converge or find an optimal path. A hybrid PSO-GA improves energy efficiency and the packet delivery ratio compared to shortest-path, PSO, and GA routing approaches in wireless sensor networks [38]. The PSO-GA protocol addresses the limitations of traditional shortest-path and minimal energy depletion routing approaches, which can lead to unbalanced energy consumption and early network partitioning. The fitness function for a particle is computed based on the maximum lifetime of a cluster head.

Another bio-inspired energy-efficient CH selection algorithm using Artificial Bee Colony (ABC) optimisation was proposed by the authors of [39]. This is a centralised approach where the BS performs all the calculations to compute the CH selection using fuzzy C-means clustering. It is considered that BS has infinite energy. The proposed method uses the node’s remaining energy, density, and location to determine the cluster head. To improve intra-cluster communication, a polling control method based on busy/idle nodes was introduced, which can reduce energy consumption and improve overall network throughput. The result showed that the optimal number of clusters improves network performance, but only for the single hop to communicate with the CH to sink.

Besides bio-inspired metaheuristic optimisation, an artificial intelligence-based clustering and routing mechanism has been studied in the literature to identify and select an optimal CH and route to the sink. A WSN can be presented as a graph, where sensors represent the nodes of the graph and their communication with other nodes forms the edges of the graph. The authors of [40] provided a comprehensive review of graph-based deep learning models for various communication methods, including wired, wireless, and software-defined networks. The survey highlighted the use of graph convolutional networks (GCNs) and graph attention networks (GANs) for wireless link scheduling, resource allocation, and network flow optimisation. Furthermore, graph deep-embedding clustering and matrix decomposition can optimise sensor deployment in large complex networks [41]. A graph auto-encoder coupled with a graph attention network has been proposed to learn the embedded representation of sensor nodes, which can then be used to partition the large-scale graph into several subgraphs. Then, singular-value-QR decomposition is used to allocate nodes within each subgraph. Using a GCN, user-aware clustering with security resource allocation was proposed in [42]. The clustering is computed based on user coverage so that all sensors are covered by at least one CH, and the process runs iteratively until all nodes are covered. However, as it focuses on user security, the energy optimisation of a node is not considered. In [43], a hybrid energy-efficient clustering was proposed using a convolutional neural network (CNN) and a modified K-means (MKM) clustering algorithm. The CNN identifies the best partitioning or cluster formation, while MKM selects the CH. ML- and DL-based models are computationally complex, requiring significant processing power and memory, which are often limited in sensor nodes. This leads to increased energy consumption, a critical issue since WSN nodes typically rely on limited battery power.

From the above literature, it can be surmised that hybrid metaheuristic algorithms perform better than single metaheuristic models for clustering and routing algorithms in terms of energy efficiency. The BOA is based on the social foraging behaviour of butterflies, using fragrance to share information among agents while incorporating information loss. Unlike the FOA and GA, the BOA’s search mechanism includes a switch probability, balancing movements towards the best solution with random walks. The BOA gives all solutions an equal opportunity for improvement, avoiding the premature discarding of potential solutions. Moreover, the BOA’s mechanisms for maintaining a balance between exploration and exploitation contribute to its robustness and stability in finding optimal clustering solutions. This helps achieve consistent results across different runs. The global search capability of PSO stands out among other metaheuristic algorithms. PSO excels at exploring the global search space to find optimal or near-optimal solutions. In routing problems, this capability is crucial for identifying the best routes among a multitude of possible paths. Additionally, PSO quickly converges to good solutions by moving particles towards the best-known positions, which is beneficial in routing problems where timely solutions are critical. No research has been conducted using a hybrid model of the BOA and PSO for clustering and routing algorithms. The performance of the BOA and PSO algorithm is highly dependent on the fitness function. In this study, we consider fitness parameters that are best suited for a disaster scenario. This study uses the hybrid model to measure the performance of the network.

## 3. Network Model

**Figure 2:**
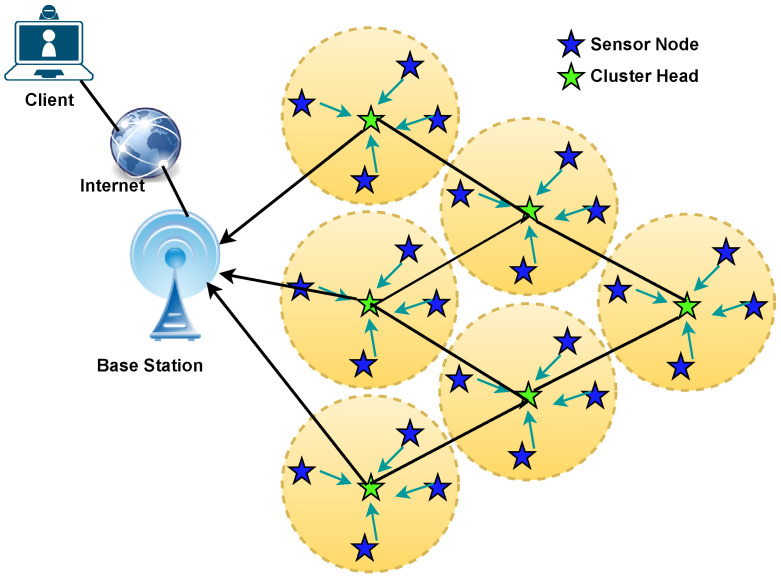
Cluster-based sensors in a WSN.

The network model used in this study is shown in Figure 2 and is based on the following assumptions:

1. The network is considered a homogeneous network. At the time of IoT-based sensor deployment, all the nodes are isomorphic, which means that all the nodes have the same energy at the time of deployment.
2. The sensor nodes are powered by batteries, and no energy-harvesting methods are applied or replenished.
3. The area covered by the IoT-based sensors is randomly distributed. The locations of the sensors are fixed, and each sensor node has a unique network identifier.
4. Each sensor node perceives its own location. The distance to other nodes can be calculated using the Euclidean distance equation.
5. The base station has unlimited energy and computational power.

**Figure 3:**
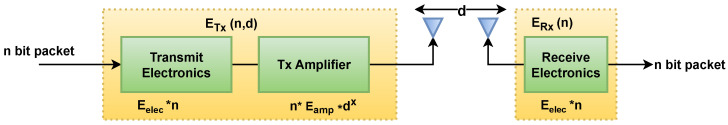
First-order radio energy model.

The first-order radio model is considered in this study, as presented by the authors of [44]. The ratio of the energy consumption model is based on the distance between the sender and receiver. Figure 3 shows the energy consumption model, where the energy required to send a message is calculated based on the sender–receiver distance and the total length of the message.

(1) $$E_{Tx}\left(n,d\right)=\left\{\begin{array}{c} n*E_{elec}+n*\varepsilon_{fs}*d^{2},\;d\leq d_{0}\\ n*E_{elec}+n*\varepsilon_{mpf}*d^{4},\;d>d_{0} \end{array}\right.$$

In Equation (1), $E_{Tx}\left(n,d\right)$ is the energy required to transmit *n* bits over the distance *d*, and $E_{elc}$ is the energy required to transmit a bit. $\varepsilon_{fs}$ is the amplification factor for the free-space model, and $\varepsilon_{mpf}$ represents the multipath fading model. $d_{0}$ is the distance threshold, defined as $d_{0}=\sqrt{\frac{\varepsilon_{fs}}{\varepsilon_{mpf}}}$. When the distance is less than the threshold, the free-space model is used, and when the distance is greater than the threshold, the multipath fading model is used. In addition, to receive *n* bits of data, the energy required is $E_{RX}=E_{elec}*n$.

## 4. Cluster Head Formation

The butterfly uses chemo-receptors to sense fragrance. While the butterfly moves randomly, it spreads intense fragrance labels. This helps the butterfly agent search for other butterflies and find a mating partner. If any given butterfly fails to detect intense fragrance, it searches randomly within the area, known as local exploitation. If the butterfly detects intense fragrance, it moves toward the source, known as global exploration. The fragrance of a butterfly can be defined as $f=CI^{\alpha }$, where *f* is the fragrance emitted by a butterfly, *c* is the sensory modality range [0, 1], *I* is the intensity of the fragrance, and α is the power exponent. Equations (2) and (3) represent the local search and global search [45]

(2) $$x_{i}^{t+1}=x_{i}^{t}+\left(r^{2}Xx_{j}^{t}-x_{k}^{t}\right)Xf_{i}$$

(3) $$x_{i}^{t+1}=x_{i}^{t}+\left(r^{2}Xg^{*}-x_{i}^{t}\right)Xf_{i}$$

where $x_{i}^{t}$ is the solution vector for the *i*-th number of butterflies at the *t*-th iteration, *r* is a random number in [0, 1], $x_{j}^{t}$ and $x_{k}^{t}$ are the positions of the *j*-th and *k*-th butterflies, and g* is the best solution in the current iteration. In our network, each butterfly is initialised with an ID ranging from 1 to *N*, where *N* is the total number of sensors. Every butterfly’s position is denoted by (Bi,p), where 1 ≤ *p* ≤ *O* and *O* is the optimal number of CHs.

Assume that *N* is the total number of sensors in the A(axa) area. Considering the free-space energy consumption model, the energy consumption of a CH per round is denoted by

(4) $$E_{CH}=n*E_{elec}\left(\frac{N}{O}-1\right)+n*E_{DA}*N+n*E_{elec}+n*\varepsilon_{fs}*d_{bs}^{2}$$

The energy consumption of a cluster member per round is denoted by

(5) $$E_{m}=n*E_{elec}+n*\varepsilon_{fs}*d_{CH}^{2}$$

The energy consumption of the cluster per round is denoted by

(6) $$E_{CPR}=E_{CH}+\left(\frac{N}{o}-1\right)*E_{M}$$

The total energy consumption of the network for the *O* number of CHs per round is denoted by

(7) $$E_{total}=E_{CPR}*O=n\left(2N*E_{elec}+N*E_{DA}+\varepsilon_{fs}\left(O_{BS}^{2}+N*d_{CH}^{2}\right)\right)$$

The optimal number of CHs can be found by differentiating *O* as denoted by [39]

(8) $$O_{opt}=\sqrt{\frac{N*n}{2\pi d_{BS}^{2}}}$$

Similarly, in the multipath fading model, the optimum number of CHs is denoted by

(9) $$O_{opt}=\frac{\sqrt{N}}{\sqrt{2}\pi }\sqrt{\frac{\varepsilon_{fs}*n}{\varepsilon_{mp}*d_{B}S^{4}}}$$

### Fitness Function

The cluster head (CH) selection is based on the residual energy ($R_{S}$), node distance $N_{D}$, and network coverage ($N_{C}$).

Residual energy: Residual energy is one of the vital factors in selecting a CH, as a CH requires more energy to perform tasks. The CH is responsible for receiving data from the node member and passing it to another CH or the BS. Therefore, the node with higher residual energy has a high chance of becoming a CH.

(10) $$R_{S}=\sum_{i=1}^{O_{opt}}\frac{1}{E_{CH_{i}}}$$

In Equation (10), $E_{CH_{i}}$ is the residual energy of the *i*-th cluster.

Node Distance: This is also an essential factor in energy consumption. The shorter the distance between the CH and a node, the less energy is required for transmission. The equation for the distance between the node and the CH is

(11) $$N_{D}=\sum_{j=1}^{O_{opt}}(\sum_{i=1}^{N_{j}}distance\left(CH_{j},X_{i}\right)/I_{j})$$

Network coverage: Higher network coverage is achieved by assigning a CH to a node that is not covered by any other CH. This provides higher scalability and increases the lifetime of the network [46]. Network coverage is denoted by [36]

(12) $$N_{C}=\frac{\left(N*-D\right)-\sum_{D=1}^{D}C*M_{D}}{\sum_{D=1}^{D}C*M_{D}}$$

The fitness function used for the BOA in this study is as follows:

(13) $$Fitness_{CH}=R_{S}*\beta 1+N_{D}*\beta 2+N_{C}\beta 3$$

where $\sum_{i=1}^{3}1,\beta_{i}\in \left(0,1\right)$. In this study, we assume β1 is 0.50 , β2 is 0.30, and β3 is 0.20.

The optimal CH selection process is given in Algorithm 1.
**Algorithm 1** CH selection algorithm process using the BOADefine the objective function using Equation (13)Create a population of butterfliesStimulus sensor modality *c*, switch probability *p* and power exponent α**while** iteration < maximum **do**      **for** each butterfly in population: **do**            calculate fragrance using            update optimal butterfly population      **end for**      **for** each butterfly in population: **do**            generate *r* (random number [0, 1])            **if** *r* < *p* **then**                 move towards best butterfly using Equation (3)            **else**                 move randomly using Equation (2)            **end if**      **end for**     update power exponent value α**end while**the optimal solution for selecting the CH

## 5. Routing Protocol

The cluster head (CH) collects data from the cluster member and sends the data to the BS. We assume that the CH maintains a list for the BS route. The main objective of this routing algorithm is to minimise the overall distance travelled from the source to the destination, as the distance is proportionally related to the energy decay and network lifetime. The fitness function evaluates the quality of the routing protocol.

In this study, we consider an improved PSO algorithm for the routing protocol. The PSO algorithm [28] is a bio-inspired method based on a swarm of birds or fish moving in a multidimensional space to search for food. Each CH node considers each of the particles, and each CH is initialised with a random velocity and position.

Let $P_{i}=P_{i,1},P_{i,2},P_{i,3},\ldots \ldots P_{i,D}$, where *D* is the optimal number of CHs. The particles update their positions based on the intensity of movement and the deviation. Particles observe $\left(L_{best}\right)$ and $\left(g_{best}\right)$ and then adjust their positions and velocities as follows [28]:

(14) $$V_{i}^{t+1}=\omega *V_{i}^{t}+C1*rand1*\left(L_{best}-P_{i}^{t}\right)+C2*rand2*\left(g_{best}-P_{i}^{t}\right)$$

(15) $$P_{i}^{t+1}=P_{i}^{t}+V^{t+1}$$

where *t* is the current iteration, ω is the inertia weight with $\omega^{max}=0.9$ and $\omega_{min}=0.2$. rand1 and rand2 are random numbers in the range [0, 1].

Each CH is assigned a random number in [0, 1] from a uniform random distribution. This number is then used to connect the CH with a random destination, known as the nearest relay node. We assume that each CH is connected within the range of either another CH or the BS. The $P_{i},D$ is assigned to the final destination node $CH_{D}$, which is connected with the BS. This process ensures that the data sent from the source CH $CH_{S}$ are routed to the BS via $CH_{D}$, thereby optimising the routing process.

The improved fitness function for the PSO algorithm considers the residual energy, the distance between the source and the destination, and the total number of relay nodes. The fitness function is as follows:

(16) $$Fitness_{PSO}=\beta 1*\sum_{i=1}^{D}Distance\left(CH_{S},CH_{d}\right)+\beta 2*\frac{\sum_{i=1}^{D}E_{r}}{avgE}+\beta 3*\sum_{i=1}^{D}relay\left(CH_{S},Dest_{d}\right)$$

where $E_{r}$ is the residual energy, AvgE is the average energy of all alive nodes, and $Distance(CH_{S},CH_{d})$ is the distance between the source and destination nodes.

**Figure 4:**
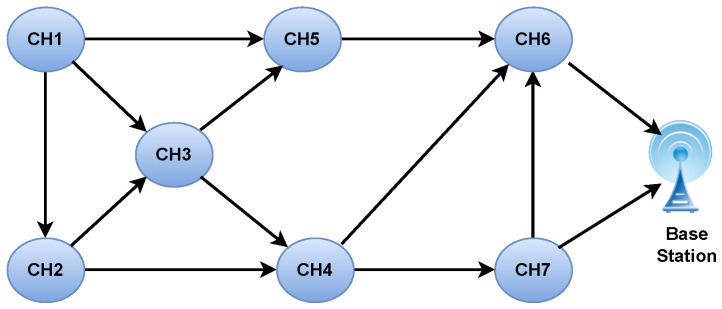
WSN topology for 5 cluster heads and one BS.

Let us consider the network shown in Figure 4, where graph G(V,E) shows the vertices, with each CH acting as a gateway, and *E* represents the edges. Table 2 shows nodes within the range of a particular CH and the total number of relay nodes. According to Figure 4, there are seven CHs (CH1,CH2,CH3,…,CH7) connected with each other within the communication range. We assign each CH a random number P(i,d)ϵ[0, 1], which maps to a random relay node destination. Some of the CHs are connected to the BS. When CH1 wants to send data to the BS, it can route the data through CH2,CH3, or CH5.

After the optimisation process is completed with the fitness function, the CHs have information about their members and the different routes to communicate with other CHs. The TDMA scheduler is used to address congestion and collision issues. Each node has a dedicated slot to send data to the CHs. The cluster members send their data according to the specific time slot assigned by the CHs. The collected data are aggregated by the CHs and sent along the optimised route to the BS according to the proposed model.

After every iteration, according to Equations (14) and (15), the particle positions and velocities update with respect to $P_{bset}$ and $G_{best}$. The updated position values might fall be ≤0 or ≥1 because of algebraic addition and subtraction. To address this issue, we regulate the positions as follows [47]:

(17) $$P\left(i,d\right)=\left\{\begin{array}{cc} 1 & \mathrm{if}\;P(i,d)>1\\ min(\vec{r}) & \mathrm{if}\;P(i,d)<0 \end{array}\right.$$

where $\vec{r}$ is a vector of random numbers in [0, 1].

**Figure 5:**
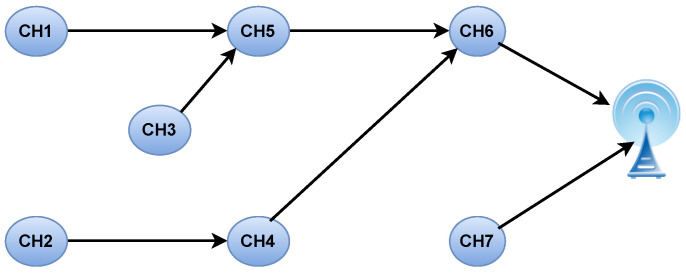
Optimisation path for the IoT network.

Table 3 shows the optimised relay paths for the network. Figure 5 shows the optimised route for each CH to aggregate data and send the data to the BS.

Algorithm 2 shows the method for selecting the optimal routing path.
**Algorithm 2** Optimal routing path selection algorithm for PSODefine the objective function using Equation (16)**Input:** Optimal number of CHs $\left(CH_{1},CH_{2},CH_{3},\ldots \ldots ,CH_{N}\right)$Initialise particles $\left(P_{i}\right)$Initialise swarm $S_{n}$**for** i=1 to $S_{n}$ **do**     compute fitness value using Equation (16)     $L_{best}$←$P_{i}$**end for****while** iteration>Maximum **do**     **for** i=1 to $S_{n}$ **do**          update the velocity and position of each particle          Evaluate the value of $P_{i}$ using Equation (16)          **if** $L_{best}>currentvalue$ **then**                $L_{best}\leftarrow P_{i}$          **else** $G_{best}\leftarrow L_{best}$                select the best fitness value          **end if**     **end for****end while**Optimal routing path by finding the optimal relay node

## 6. Time Complexity Analysis

### 6.1. Hybrid BOA-PSO Time Complexity

The time complexity of the hybrid BOA-PSO depends on the following factors:

- Clustering Time Complexity: The time complexity of the BOA depends on the population size N, the fitness function F(x), and the dimension of the area D. The time complexity for the dimension of the area is O(D), and for the fitness function, it is O(F(x). The total time complexity of the BOA is O(D+F(x).
- Routing Time Complexity: The time complexity of the PSO routing algorithm depends on various factors, such as the particle’s initial position, velocity, update position, and number of iterations required to complete the routing path. The time complexity of initialising the particles is O(N). The update rules for particle velocities and positions also have a time complexity of O(N). The fitness of each particle involves calculating the cost of a route or path, which can be evaluated by F(x). The time complexity to evaluate a route for a single particle is O(F(x)). For *N* particles, this becomes O(N.F(x)). For *I* iterations, the time complexity can be written as O(I.(N.f(x)+N).

The total time complexity of the hybrid BOA-PSO algorithm is *O*(*D* + *F*(*x*) + *O*(*I*.(*N*.*f*(*x*) + *N*).

### 6.2. LEACH Structure and Time Complexity

The LEACH protocol is characterised by a distributed approach, where nodes make local decisions without central control. It is designed for energy efficiency, aiming to conserve energy and extend network lifetime by minimising communication and balancing the load across nodes. LEACH effectively manages scalability through clustering, making it suitable for large networks. However, it assumes that all nodes have homogeneous energy levels and does not account for heterogeneous networks with varying energy capacities. The time complexity of the LEACH algorithm depends on the following factors:

- Initialisation: The time complexity depends on the initial distribution of the number of nodes n, and ranges from O(nlogn) to $O(n^{2}).$
- Local Exchange: The local exchange of information to search for the nearest neighbour requires $O(n^{2})$ per iteration.
- Total Iterations: For I number of iterations, the time complexity is O(I)

The total time complexity of the LEACH algorithm is $O(I.n^{2}(nlog\left(n\right)$.

### 6.3. DEEC Structure and Time Complexity

The DEEC protocol selects cluster heads probabilistically based on the residual energy of nodes, with higher-energy nodes more likely to be chosen. It operates in a distributed manner, with local decisions for cluster formation and cluster head selection. DEEC functions in rounds, where each round includes cluster head selection, cluster formation, data collection, and transmission. After each round, the system resets, and the process repeats based on the updated energy levels and network conditions. The structure of the DEEC algorithm is as follows:

- Cluster Formation: The nodes form clusters based on energy levels. Each node evaluates its potential as a cluster head and communicates with its neighbours. This process typically involves a series of messages between nodes, and the time required is $O(n^{2})$.
- Cluster Head Selection: Neighbour nodes decide whether to become cluster heads based on their energy levels and other criteria. This process involves evaluating and comparing energy levels across all nodes, which takes O(n) time for each node.
- Data Aggregation and Communication: This involves collecting and aggregating data within clusters. For K clusters with m nodes per cluster, this has a complexity of O(K.m).

The total time complexity for I iterations is $O(T.\left(k.m\right).n^{2}$.

## 7. Simulation Results

A simulation was conducted to validate the proposed model. Matlab 2019b was used to perform the simulation. The properties of the Matlab installation environment were as follows: Windows 10 64-bit operating system, Intel(R) Core(TM) i7-6700 CPU @ 3.40 GHz, and 16 GB of RAM. To validate the model, the simulation results of the proposed model were compared with those of the traditional LEACH model [44], DEEC model [48], a state-of-the-art metaheuristic algorithm based on PSO [35] and hybrid PSO-GA [38] and PSO-HSA [36] models. The parameters used for this simulation are given in Table 4.

The simulation considered two scenarios for a 200 × 200 m area. Based on the literature [34,36,39], we identified that choosing a 200 × 200 m simulation area for WSN applications in environmental monitoring balances computational efficiency and practical considerations. Smaller areas require less computational power and allow for faster iterations, which aids in resource management and the testing of various configurations. Moreover, we used it as an early-stage research and proof of concept for the proposed method, providing a controlled environment for baseline comparisons before scaling up. Additionally, larger areas introduce more variability and complexity in propagation models, while smaller areas help validate models and algorithms before addressing scalability issues.

**Scenario 1**: This scenario represents a normal situation, where the BS/sink is located at (100,100). All the sensors are positioned around the BS for short-range communication.

**Scenario 2**: This scenario represents a disaster situation, where the BS/sink is located at (0,0). All the sensors are situated far from the BS for long-range communication.

This simulation helps to understand and compare the proposed model’s stability under different conditions with different models.

The following performance metrics were considered to validate the proposed model:

- Residual Energy: The sum of all the remaining energy of all alive sensor nodes was used as a performance metric to evaluate the model. Residual energy is directly related to energy utilisation within the network and influences the network’s lifetime.
- Throughput: This is another important performance factor for evaluating the protocol. Throughput defines how much information the client receives from the monitoring area that the sensors collect and send. It measures how many packets the BS receives from the sensor nodes.
- Network lifetime: The main objective of the proposed model is to maximise network lifetime. This metric measures how many nodes are alive and able to send information to the sink. As mentioned above, this metric is directly related to the residual energy of a node.

**Figure 6:**
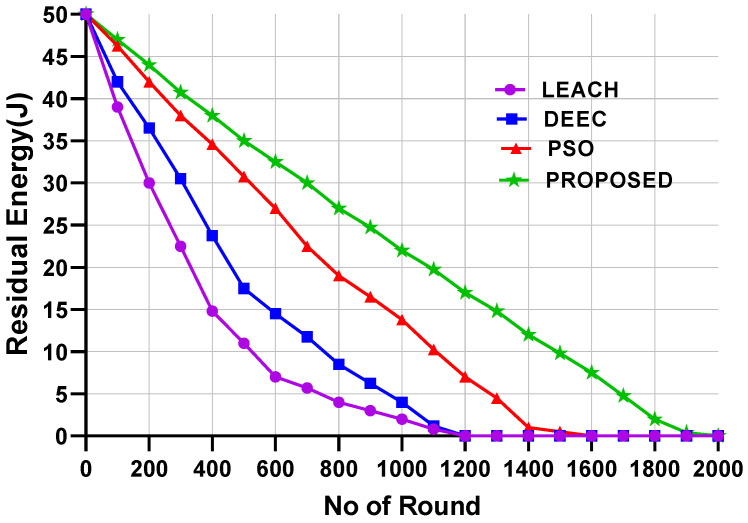
Residual energy for Scenario 1.

**Figure 7:**
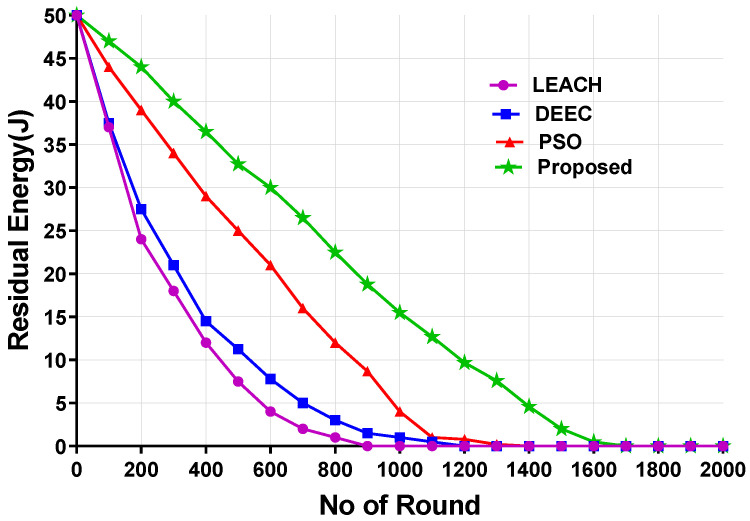
Residual energy for Scenario 2.

Figure 6 and Figure 7 present the average residual energy of all nodes for Scenario 1 and Scenario 2, respectively. The residual energy of the proposed model is compared with that of the LEACH, DEEC, and PSO models. For the calculation of the residual energy, 200 nodes were considered for both scenarios. According to the figures, the proposed model achieved the highest residual energy for all nodes across different numbers of rounds.

For Scenario 1 (Figure 6), the LEACH and DEEC models showed that all nodes’ residual energy was depleted after 1200 rounds. PSO performed better than the LEACH and DEEC models, with residual energy becoming low around 1450 rounds. However, the proposed model performed much better than the LEACH and DEEC models, with the residual energy lasting until after 1957 rounds.

On the other hand, for Scenario 2, the residual energy for LEACH, DEEC, and PSO depleted at 967, 1187, and 1325 rounds, respectively. However, the proposed model lasted until 1600 rounds. The LEACH model experienced higher energy consumption due to the random selection of CHs and single-hop data transmission. For the DEEC algorithm, the optimal distance is not considered when selecting CHs. The PSO model considers the residual energy and distance to the BS but does not consider the distance between CHs and orphan nodes. The proposed model performed better as it considers higher residual energy, distance, and orphan nodes, thereby extending the network’s lifetime.

**Figure 8:**
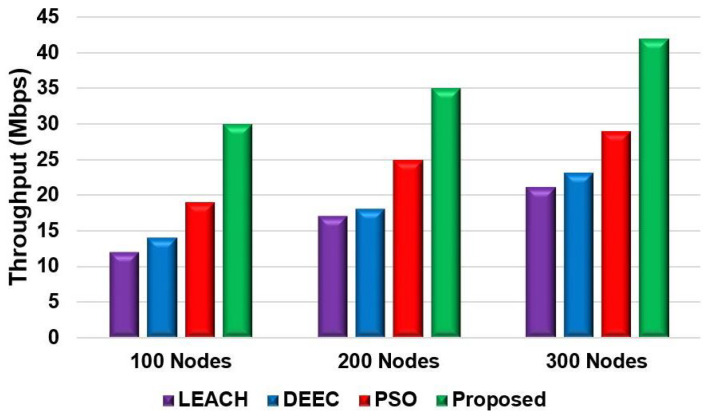
Throughput comparison of different nodes for Scenario 1.

**Figure 9:**
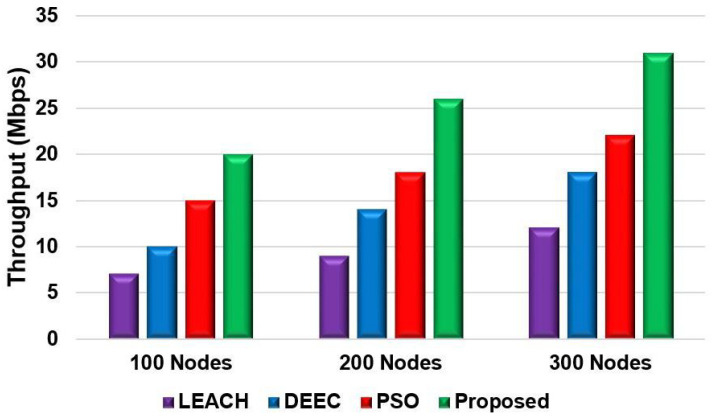
Throughput comparison of different nodes for Scenario 2.

Figure 8 and Figure 9 show the network performance throughput for the proposed model compared with that of the LEACH, DEEC, and PSO models for Scenario 1 and Scenario 2, respectively. The throughput was also measured by changing the number of nodes. In both scenarios, the proposed model’s network throughput was significantly higher than that of the other protocols. The proposed model achieved a throughput increase of 10 to 20% compared to the other models. This can be attributed to the proposed model’s fitness function, which reduces energy consumption during data transmission to achieve a higher network throughput.

**Figure 10:**
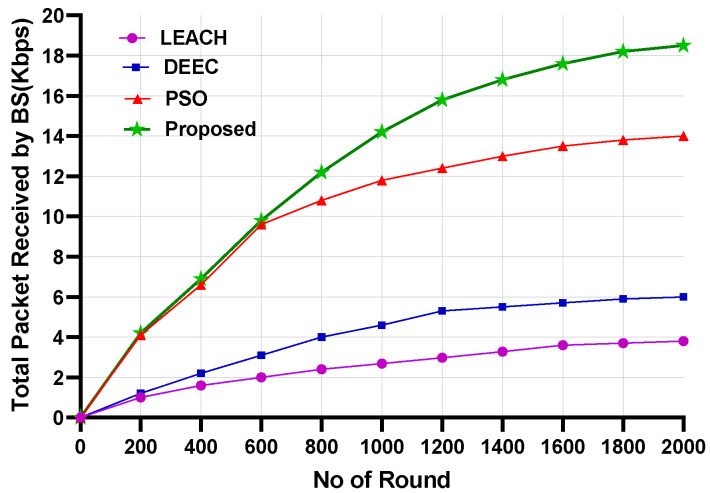
Number of packets received by the BS.

**Figure 11:**
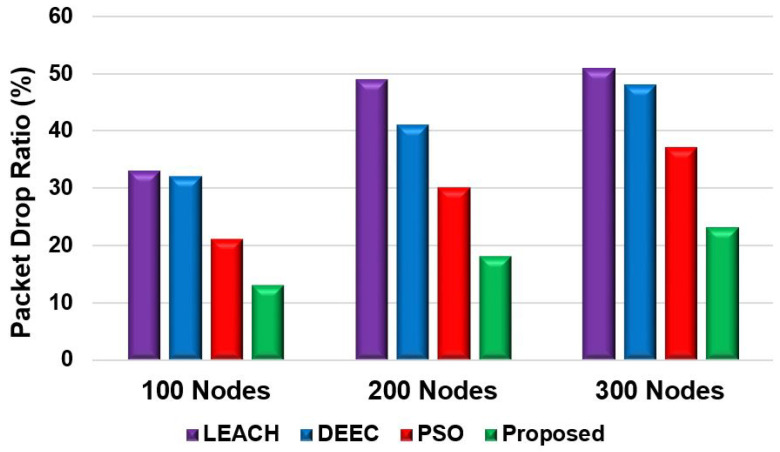
Packet-drop ratio for different numbers of nodes in a WSN.

**Figure 12:**
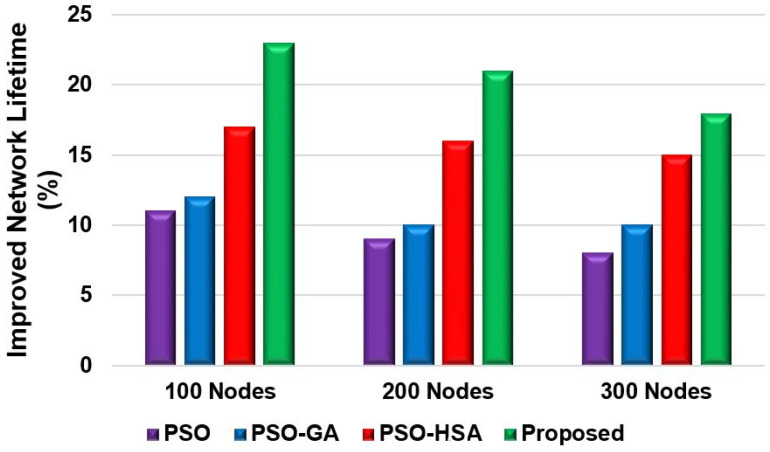
Percentage of improved network lifetime for Scenario 1.

**Figure 13:**
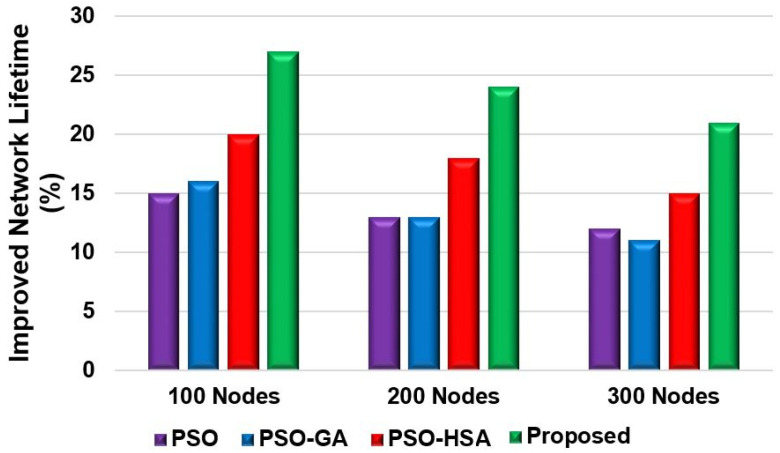
Percentage of improved network lifetime for Scenario 2.

Figure 10 shows the total packets received by the BS. Scenario 2 considered 200 nodes for this evaluation. More than 18,000 packets were received by the BS for the proposed model. However, the LEACH and DEEC models could not send as many packets compared to the PSO model. The proposed model sent over 12,000 more packets than the LEACH and DEEC models and 4000 more packets than the PSO model. The reason for this is that the proposed model’s fitness function preserves the nodes’ residual energy, which extends their lifetime. This enhances network lifetime and increases the number of packets transmitted to the BS. Figure 11 shows the packet-drop ratio for Scenario 2 with varying numbers of nodes. The proposed model achieved a lower packet-drop ratio compared to the other models. The LEACH and DEEC models had significantly higher packet-drop ratios for different node numbers compared to the proposed model. The packet-drop ratio for the proposed model increased by approximately 5% with the addition of 100 nodes. The proposed fitness function helps minimise data transfer drops as the model selects energy-efficient paths to send the data. However, the PSO model achieved a lower drop ratio because of the routing efficiency. Nevertheless, due to the inefficient selection of CHs and routing paths, the LEACH and DEEC models achieved higher packet-drop ratios. Figure 12 and Figure 13 show the percentage improvement in network lifetime for the proposed model in Scenario 1 and Scenario 2, respectively. The proposed model is compared with the state-of-the-art PSO and hybrid models. The proposed model significantly improved network lifetime, increasing by 27%, 24%, and 21% in Scenario 1 for 100, 200, and 300 nodes, respectively. In Scenario 2 (Figure 13), the proposed model achieved increases of 23%, 21%, and 17% for 100, 200 and 300 nodes. The proposed model achieved a higher network lifetime compared to the other state-of-the-art models, with the network lifetime increasing by approximately 10%. This is due to the efficient CH selection process, which reduces node energy consumption during packet transmission and finds the optimised route for packet delivery.

## 8. Conclusions and Future Directions

This paper proposes a bio-inspired optimisation algorithm model for selecting energy-efficient cluster heads and the optimal routing protocol. The proposed model aims to increase network lifetime and reduce energy consumption, which are vital parameters in an IoT network during a disaster. Effective fitness functions for CH selection and routing are used to enhance the model’s performance. The analysis considered different scenarios by placing the BS in different locations. The proposed model was compared and evaluated against benchmark models that use CH-based routing protocols for IoT networks. The performance metrics used to evaluate the proposed model included residual energy, network throughput, packet delivery, and network lifetime. The experimental results showed that the proposed model significantly enhanced residual energy by more than 10% in both scenarios across all the considered protocols and achieved 60% higher throughput than the LEACH model, 53% higher throughput than the DEEC model, and 37% higher throughput than the PSO protocol. In addition, network lifetime increased by 10–20% compared to the LEACH, DEEC, PSO, and hybrid PSO models.

In the future, we will consider more comprehensive scenarios when extending our simulation models. It is also necessary to validate the proposed model against a broader range of bio-inspired models. To calculate energy consumption for cluster formation and CH selection, we will extend our model with more realistic propagation models, such as the Hata model, extended Hata model, Hata–Okumura model, and Ericson model, in rural, suburban, and urban areas. Our future research will address the challenges posed by rapid and unpredictable changes in network topology during disaster scenarios. Therefore, developing algorithms that can dynamically adapt to node mobility and ensure continuous communication is essential. In addition, we will explore specialised clustering algorithms for heterogeneous networks and investigate the application of machine learning and AI to adaptive clustering to improve network performance in disaster scenarios. In this research, we simulated the proposed method in a small area. In our future work, we will expand the areas and incorporate higher sensor density and complex propagation models.

## Figures and Tables

**Table 1:** The list of the acronyms used in this research.

Abbreviation	Definition
ABC	Artificial Bee Colony
ACO	Ant Colony Optimisation
BA	Bat Algorithm
BBO	Biogeography-Based Optimisation
BOA	Butterfly Optimisation Algorithm
BOA-PSO	Butterfly Optimisation Algorithm-Particle Swarm Optimisation
BICR	Bio-Inspired energy-efficient Cluster and Routing
BS	Base Station
CH	Cluster Head
CNN	Convolutional Neural Network
CSA	Cuckoo Search Algorithm
DEEC	Distributed Energy-Efficient Clustering
EWS	Early Warning System
FOA	Firefly Optimisation Algorithm
GA	Genetic Algorithm
GAN	Graph Attention Network
GCN	Graph Convolutional Network
HBMO	Honey Bee Mating Optimisation
HSA	Harmony Search Algorithm
IoT	Internet of Things
LEACH	Low-Energy Adaptive Clustering Hierarchy
LOA	Lion Optimisation Algorithm
LPO	Lion Pride Optimiser
PCA	Principal Component Analysis
PIO	Pigeon-Inspired Optimisation
PSO	Particle Swarm Optimisation
PSO-GA	Particle Swarm Optimisation-Genetic Algorithm
PSO-HSA	Particle Swarm Optimisation-Harmony Search Algorithm
RFID	Radio-Frequency IDentification
RIO	Roach Infestation Optimisation
TDMA	Time-Division Multiple Access
WSN	Wireless Sensor Network

**Table 2:** Possible relay nodes of the CHs.

Cluster Head	Relay Node	No. of Relay Nodes
CH1	{CH2, CH3, CH5}	3
CH2	{CH3, CH4}	2
CH3	{CH4, CH5}	2
CH4	{CH6, CH7}	2
CH5	{CH6}	1
CH6	{BS}	1
CH7	{CH6, BS}	2

**Table 3:** Optimised relay paths of the CHs.

Cluster Head	Relay Node	No of Relay Node	P(i,d)	Relay Node
CH1	{CH2, CH3, CH5}	3	0.65	CH5
CH2	{CH3, CH4}	2	0.73	C4
CH3	{CH4, CH5}	2	0.96	C5
CH4	{CH6, CH7}	2	0.52	CH6
CH5	{CH6}	1	0.87	CH6
CH6	{BS}	1	0.29	BS
CH7	{CH6, BS}	2	0.18	BS

**Table 4:** Possible relay nodes of the CH.

Parameter	Value
Initial energy $E_{o}$	0.5 j
Transmission and receive energy $\left(E_{elec}\right)$	50 (nj/bit)
Multipath fading $\left(E_{amp}\right)$	0.0013 pj/bit/m^4^
Free-space transmitter amplifier energy $\left(E_{fs}\right)$	10 pj/bit/m^2^
Power exponent	0.1
Sensory modality	0.01
Particle position $\left(X_{min},X_{max}\right)$	0,200
Particle velocity$\left(V_{min},V_{max}\right)$m/s	0,200
No. of rounds	2000
No. of iterations	5
Swarm size	15
Acceleration constant	2

## Data Availability

Data are contained within the article.
